# Learning from the Drug Policy Curriculum: People Who Use Drugs Policing in the Context of Decriminalization

**DOI:** 10.1177/00914509251396996

**Published:** 2025-11-20

**Authors:** Naomi Zakimi, Becca Wood, Rodrigo Santos, Alissa Greer

**Affiliations:** 1 School of Criminology, 1763 Simon Fraser University, Burnaby, British Columbia, Canada; 2 Faculty of Education, 141631 University of British Columbia, Vancouver, British Columbia, Canada

**Keywords:** drug policy, decriminalization, drugs policing, curriculum, social position

## Abstract

Police officers have long been tasked with translating drug policies into practice; as a key public-facing side of the criminal justice system, they influence how drug policy messages are conveyed to the public through everyday enforcement practices. The government of British Columbia, Canada, received a 3-year exemption from federal drug laws to decriminalize the possession of small amounts of most illicit substances starting January 31, 2023. In this context, we explored what people who use drugs learned from drug policy as it was taken up into policing practice. We use constructs from curriculum theory as a framework to understand what policing explicitly and implicitly communicates to people who use drugs. We analyzed 40 qualitative interviews with people who use drugs in socioeconomically stable positions (housed and employed) in the first year of decriminalization in British Columbia to understand lessons gleaned from policy and policing in this policy context. Findings show that the formal curriculum of drug policy provided a sense of relief for many participants who could ease their fears of being labelled “criminals.” However, the way that drug policies were applied by officers in practice, making explicit a hidden curriculum, shaped how participants saw themselves and other people who use drugs in ways that were stigmatizing. Our research shows the value of analyzing the hidden curriculum of drug policy to illuminate how it shapes the way in which people who use drugs construct and position themselves.

## Introduction

The role and impact of police officers in the lives of people who use drugs is a contentious and politicized issue. Regardless of how we assess the utility and necessity of police officers in illicit drug markets, they continue to play a role in the enforcement of drug policy throughout most of the world. In Canada specifically, the role of police is highly contested amid shifting drug policies that aim to address the drug toxicity crisis, such as the Good Samaritan Drug Overdose Act, which provides legal protections for drug possession to people at overdose events, and, most recently, decriminalization in British Columbia (Government of British Columbia, 2022; Government of Canada, 2021; Latimore & Bergstein, 2017; Zakimi et al., 2022). Police officers undeniably play a pivotal role in taking up such drug policies as they are tasked with understanding, translating, and enforcing them (or not) in day-to-day policing practice.

When it comes to policing, research has shown that social positioning among people who use drugs plays a key role in determining who is criminalized. There is a burgeoning literature on people who use drugs’ perspectives of and experiences with police officers in different contexts, which shows that unhoused, racialized, and sex workers are disproportionately subjected to police contact, violence, and harassment (Beletsky et al., 2015; Bungay et al., 2010; Friedman et al., 2021; Greer et al., 2021; Sarang et al., 2010; Selfridge et al., 2020; van der Meulen et al., 2021; Werb et al., 2008; Wood et al., 2017; Xavier et al., 2022). However, a growing body of research has also explored the nuanced and diverse experiences of people who are involved in the illicit drug market (use and/or sell drugs) across the socioeconomic spectrum, including those who have relatively less contact with the criminal justice system (Askew & Salinas, 2019; Berger, 2025; Perrin et al., 2021; Rodner, 2005).

Yet, little is known specifically about how people who use drugs who are less likely to come into contact with the criminal justice system (such as people who use drugs in “party” or “recreational” contexts or White middle-class users) view and experience policing, especially in the context of decriminalization or other drug liberalization policies. Previous research on “socially included” people who use drugs and “recreational” users suggests that they are protected from formal legal sanctions due to their ties to mainstream society and ability to conceal drug use (Askew & Salinas, 2019; Cruz, 2015; Hausser et al., 1999). Given that policing holds symbolic meaning for the public as a whole, shaping understandings of identity and group belonging (Loader & Mulcahy, 2003), the perspectives of people who use drugs who are less vulnerable to policing can enrich our existing understanding of policing inequity and experiences of different drug policy regimes.

Policing plays a key role in shaping discourse, identity, and social structure amongst citizens and the community. For the public and people who use drugs specifically, police officers tend to be their first point of contact with the criminal justice system and can thus influence how people see and position themselves and others through labelling and legitimizing some individuals and groups as “dangerous” (Justice & Meares, 2014; Loader & Mulcahy, 2003). In this role, police officers can be seen as implementors of the criminal justice “curriculum” (Meares, 2016), which, in educational terms, can be defined as a plan or course of “studies” that legitimizes knowledge and messages about identity, belonging, and social structure (Apple, 1971). In other words, police officers are part of a criminal justice “curriculum” and can teach knowledge and values present in drug policy to the people they interact with. Policing can evoke images, feelings, and perceptions of oneself, others, and the world; it is one way that we make sense of our world (Loader & Mulcahy, 2003).

The current study focuses on the views and experiences of a sample of people who use drugs who were “socioeconomically stable” (housed and employed) and, ultimately, had less frequent police contact. We analyze qualitative data from this sample in the year following decriminalization in British Columbia to explore how this policy operates as a “curriculum” through policing practices, and how these practices shape and limit how participants see and position themselves as people who use drugs. By focusing on this often-overlooked demographic, the research contributes to a more nuanced understanding of people who use drugs’ views and experiences, highlighting the role of both drug policy and law enforcement in shaping how people see and position themselves within broader social structures. Through this research, our aims are not to categorically define people who use drugs or claim some people's drug use as “normal” and others’ as criminal; rather, we focus on the “how”—how can drug policy produce some people who use drugs as criminal but not others (Dertadian & Askew, 2024), even in a context that decriminalized drug use.

In the following sections, we first review the literature to illuminate how policing interacts with social position and then expand on how policing, in its role as interpreters and enforcers of the official drug policy “curriculum,” can influence how people who use drugs see and position themselves.

### People Who Use Drugs, Social Position, and Inequity in Policing

The effectiveness of drug policies is shaped by how they are understood and translated into practice. This perspective emphasizes the pivotal role that police officers play in realizing the impact of policies and their aims. That is, policing can create a gap between the intent of drug policies and what is practiced or experienced. The way that drug laws are enforced “on the street” can be different from the law “on the books” (Burris et al., 2010). Studies suggest that police interactions with people who use drugs are shaped by intersecting socioeconomic factors, such as housing status, income, race or ethnicity, and gender. Qualitative and quantitative research shows that people who use drugs who are unhoused, racialized, or sex workers are disproportionately subjected to police violence (Bungay et al., 2010; Friedman et al., 2021; Sarang et al., 2010; Selfridge et al., 2020; Werb et al., 2008). Canadian research shows specifically that unhoused people who use drugs experience more frequent police interactions and harassment, often driven by factors such as open drug use and heightened visibility in public spaces (Greer et al., 2022a).

Social positioning also influences criminal justice outcomes, with marginalized people who use drugs facing disproportionately high rates of incarceration and negative sentencing outcomes. For example, in Canada, Indigenous peoples are overrepresented in the criminal justice system, both as victims/survivors and accused/convicted, due to systemic discrimination and institutional racism (David & Mitchell, 2021; Department of Justice, 2024). Therefore, existing policing and criminal justice practices put people who use drugs at risk of harms, such as increased HIV transmission and other health complications, displacement to less visible and potentially more dangerous environments, reduced access to and use of harm reduction services, heightened overdose risk, and increased exposure to violence and abuse (Aitken et al., 2002; DeBeck et al., 2017; Kerr et al., 2005).

The conventional and concealed lives of people who use drugs who are considered socioeconomically stable or “socially integrated” seem to largely protect them from formal legal sanctions due to their ties to mainstream society (Askew & Salinas, 2019; Cruz, 2015; Hausser et al., 1999; Rödner, 2006). For example, in a qualitative study conducted by Perrin et al. (2021), women who use or sell drugs ascribed their lack of criminal consequences by making reference to negative stereotypes associated with people who use drugs, such as looking “clean,” avoiding piercings or dreadlocks, and not looking tired. Further, access to private housing may reduce interactions between people who use drugs and police officers by lowering their visibility within public spaces, thereby decreasing the likelihood of surveillance and targeting (Greer et al., 2022a; Rödner, 2006). Consequently, experiences with police officers are shaped by a range of intersecting factors, including social class, gender, and race or ethnicity. These dimensions of social positioning influence how equitably the impacts of drug policy are distributed, which determines, in part, who is protected and who is penalized. In light of ongoing shifts in drug policy, it is important to examine the perspectives of people who use drugs and their diverse experiences with police officers (or lack thereof) to understand the ways in which drug policies can shape how people who use drugs see and position themselves as they are translated into practice.

### Drug Policy as Curriculum: How Policing Teaches Us About the World

Ample previous research has theorized how people who use drugs see and construct themselves, most notably through social identity and poststructural lenses. Social identity approaches, rooted in social psychology, have contributed extensively to our understanding of how people who use drugs construct and manage their sense of selves in relation to and comparison to the social groups to which they belong (Buckingham et al., 2013; Pickard, 2021; Sibley et al., 2023). Generally speaking, it proposes that people categorize themselves into social groups and, through comparisons with out-groups, evaluate the value of their groups, which ultimately shape their self-concept and a positive or negative social identity (Tajfel & Turner, 2004). Studies have shown that people who use drugs often shape their identity based on personal characteristics like drug of choice, route of administration, recovery approach, gender, or age (Sibley et al., 2023). They regularly identify with categories that allow them to distance themselves from stigmatized conceptions of drug users as “addict” or “junkie,” leading to intragroup differentiation (Sibley et al., 2023). For instance, a study with newly abstinent people who used opioids found that they refused to carry naloxone because they perceived it as mismatched with their new treatment-abstinent identity, posing a threat to their in-group belonging and acceptance by linking them to their previous drug-using identity (Bowles et al., 2021).

In drug policy scholarship, drawing on poststructuralism, people who use drugs are theorized as being constituted through specific material and discursive practices (Bacchi, 2009; Moore & Fraser, 2006). Drug policy thus shapes the subject positions, the discursively produced roles that people can occupy, such as “responsible citizen” or “dangerous drug user,” that are made available for people who use drugs (Bacchi, 2009; Ritter, 2021). Through this lens, people (or subjects) are not considered “fixed” or easily categorized, but always in flux and in-the-making. People who use drugs are often constituted through dividing practices that position some as “chaotic” or “irrational addicts” in contrast with the responsible and functioning neoliberal citizen or the “virtuous” drug user who uses drugs for spiritual or therapeutic purposes (Chatwin & Alexander, 2025; Moore & Fraser, 2006). For example, a study by Walker et al. (2020) analyzed interviews with young men who inject drugs in prison and prison policy texts and found that prohibitionist policies produced people who inject drugs in contradictory ways, both as “dirty” and “irresponsible junkies” and as “rational” actors responsible for minimizing harms, ultimately increasing harmful injection practices. As such, drug policy can limit the subject positions that people who use drugs can take on, resulting in potentially harmful lived effects.

In this study, we apply curriculum theory using an interpretive social constructionist approach. Recognizing that police officers are central actors in the enactment of drug policy, curriculum literature offers a useful lens for understanding how policies are enacted, as well as how unintended or implicit lessons may be communicated through this process. The field of curriculum theory is composed of a wide variety of perspectives and theoretical approaches (Pinar et al., 1995). In the traditional sense, “curriculum” refers simply to a plan or course of studies; it is the official educational plan governments create to guide teachers on what and when topics should be taught during a school year for a specific subject (Tyler, 1974). In the current study, we engage with a broader definition of curriculum that encompasses both the formal plans (explicit) and “hidden” (implicit) values and knowledge that are taught through experiences inside and outside of a school context (Jackson, 1992). We conceptualize curricula as political and ideological tools that allow the state to shape the kind of citizens it seeks to create, both through official documents and their translation into practice (Apple, 1971).

By examining curriculum as enacted, a “hidden” curriculum is illuminated. The “hidden” curriculum communicates unstated knowledge, influencing what students learn about their role in society and revealing underlying values and ideological messages in the official plan of studies (Apple, 1971; Giroux & Penna, 1979, p. 21; Jackson, 1968). The hidden curriculum can include what is taught tacitly by how schools are organized and how students interact with teachers. The hierarchical organization of schools, for example, communicates broader lessons about social order in the outside world (Giroux & Penna, 1979; Jackson, 1968). All the learning experiences must be taken into account. Specifically, uncovering and naming the underlying values and ideological messages in the curriculum can encourage debate and reflection among students, empowering them to decide which ideas or messages to take up (Neve & Collett, 2018) while also evidencing which discourses about schooling and education are prioritized (Bernstein, 1996).

Curriculum scholars have long moved away from conceiving the curriculum as a fixed sequence of objectives, treating it instead as a discursive construction. The curriculum is a set of policies and practices that produce meanings, values, and “truths” (Ball, 1994; Bernstein, 1996). While privileging some knowledges and marginalizing others, it normalizes and idealizes what counts as a “good” teacher or student (Popkewitz, 1998). Even when teachers resist or oppose the implementation of curriculum policies, the curriculum can still operate as a technology of regulation that reshapes not only what teachers do but also who they are (Ball, 2003). Similarly, for students, the curriculum can consolidate hegemony (Gramsci, 1971), building consent around a singular, official view of reality (Apple, 2004). If not contested in practice by teachers and students, the curriculum can form subjects oriented towards compliance with the state's view of knowledge and social order (Giroux, 1981).

A curricular document, like other policy documents, has a life (Apple, 2019) and is recontextualized and enacted across sites rather than simply implemented. It not only represents disputes among stakeholders who have succeeded in inscribing their particular views of the world, education, and society into the official text, but it is also enacted differently by different policy actors (Ball et al., 2012). In these on-the-ground translations and interactions, actors interpret and select knowledge and practices, implementing policy in their own ways, generating effects beyond what is written, and, in the process, reshaping themselves and others as subjects (Rose, 1999). Accordingly, in this paper, we follow this line of inquiry in curriculum theory, assuming that reality is constructed in subject–object interactions and that policies and subjects are co-produced in those same interactions.

These ideas from curriculum theory can provide a unique lens to examine drug policy. Recent research has proposed that the criminal justice system can be seen from an educational lens; specifically, Justice (2017) and Justice and Meares (2014) propose that the criminal justice system, including police, is a site for civic education. The justice system provides both a formal and hidden curriculum that teaches citizens about civic identity; that is, policing can send a message to people about “who is a citizen and who is a problem” (Justice & Meares, 2014, p. 162).

In the criminal justice system, police officers play a key role in knowing, understanding, and applying policy “on the books” into practice “on the street” (Burris et al., 2010; Justice & Meares, 2014). In British Columbia specifically, officers have been tasked with enacting the formal curriculum of drug policy changes in recent years (Greer et al., 2022b; Xavier et al., 2021). Through this role, officers may also be communicating information through a hidden curriculum. Police officers enact the curriculum when they arrest someone for drug trafficking or when they confiscate illicit drugs, which shows that these curricula allow police officers to limit freedoms under the stated objective of protecting the public from crime (Justice & Meares, 2014). The “hidden” curriculum is translated through officers’ actions and practices (or lack thereof) that are not outlined in the formal curriculum, but which nevertheless transmit ideas and values to the public. For instance, policing practices function to exert social control over marginalized groups of people who use drugs in alignment with neoliberal logics, even if this is not their overt or explicitly stated objective, pushing us to consider the role of stigma as politically productive (Fraser et al., 2017; Tyler & Slater, 2018; Wacquant, 2009).

Previous research illuminates the importance of messaging through policy and practice. For example, police disproportionately stop and arrest Black youth in Toronto compared to their White counterparts (Meng, 2017), and unhoused people are at higher risk of police contact (Greer et al., 2022a); these ways of enacting the law communicate information about social hierarchy and belonging. Policing practices communicate implicit lessons about social groups and identity and reinforce notions of who belongs and who is labelled as “undesirable and dangerous people different from everyone else” (Justice & Meares, 2014, p. 1530). In their study of policing in England, Loader and Mulcahy propose that police have the “power to diagnose, classify, authorize, and represent both individuals and the world, and to have this power of ‘legitimate naming’ not just taken seriously, but taken for granted” (Loader & Mulcahy, 2003, p. 226). Therefore, police officers, through their decision-making and practices, teach and communicate information about drugs and the groups people belong to through their actions and inaction. In the current study, the focus is on the people who receive such information, rather than on police officers, as well as what they learn from policing practices. Using a curriculum theory lens centres the state as the designer of a civic learning plan and is concerned with the process by which the state communicates such knowledge and people receive it (Meares & Prowse, 2021), such as through messaging about and implementation of social policies.

### Study Aims

In the current study, we suggest that police officers can “teach” or pass on knowledge to citizens about who they are and the groups they belong to through their enactment of the formal and hidden curriculum of drug policy reforms. Examining what policing teaches people who use drugs in the context of drug policy change, where police officers’ ability to charge people with simple possession is theoretically reduced, allows us to explore a rare and unique policy context where public awareness of the “curriculum” is potentially heightened. It further sheds light on the way that drug policy changes, like decriminalization, are received by people who use drugs, framing policy change not as a one-way street of communication, but as a relational process where police officers continue to play a pivotal role. Specifically, we ask how people who use drugs experience and understand drugs policing, and how the formal and hidden curricula of drug policy, enacted in police practice, shape how participants see and position themselves as people who use drugs.

## Data and Methods

Between August and December 2023, we conducted 40 semi-structured interviews with people who use drugs who were stably housed and employed in British Columbia as part of a larger study about decriminalization. We recruited this sample because it provided a lens through which to better understand the unequal weight of drug policy and drug policing on people who use drugs. Further, rather than sampling for people based on an assumption of how and why they use drugs (e.g., for “recreation,” in a “controlled” way), which leaves unquestioned the idea that these categories pre-exist individuals and reinforces the “othering” of people who do not use drugs recreationally, we make clear that we are looking at socioeconomic position and that socioeconomic stability does not necessarily mean “recreational” or “virtuous” drug use (Chatwin & Alexander, 2025), avoiding this taken-for-granted association. Remaining silent about the experiences of more privileged people who use drugs “works to maintain existing social and cultural hierarchies” (Zampini et al., 2021, p. 3). Thus, we draw attention to the role of social positioning in shaping the experiences of people who use drugs, focusing here on those who are in privileged positions relative to other people who use drugs in British Columbia who are structurally marginalized.

The eligibility requirements to participate included: being 18 years old or older; speaking English; have lived in British Columbia for the past 6 months; have used one or more of the decriminalized drugs at least once a month for the past 6 months to ensure participants could speak to the personal impact (or lack thereof) of decriminalization; have been in private non-subsidized housing for the past 6 months; and have been employed for the past 6 months (part-time or full-time, in one or multiple jobs, temporary or permanent positions). Participants were recruited from social media platforms, such as Twitter, Facebook, Instagram, LinkedIn, and Craigslist, as well as through snowball sampling, as some participants shared our study with their network. Each participant received a CAD $40 honorarium. The interview guide was developed in consultation with the research team, with multiple revisions based on previous literature, developments in the decriminalization model, and adapted following initial interviews. The final interview guide was divided into four main topic areas: decriminalization awareness and knowledge, expected or experienced impact of decriminalization, experiences with police, and health and social supports. Although decriminalization is strongly related to discussions of police, which came up organically during the interviews, we also prompted participants to discuss their views about police by asking questions such as: “Generally speaking, what are your views about police?”, “What have your drug-related experiences with police been like in the past 12 months?”, and “how do you feel about potentially interacting with police officers since drugs became decriminalized?”. As we conducted interviews and began reading and analyzing the first transcripts, we refined the interview guide to promote relevance in the current context by expanding and adapting questions that reflected participants’ experiences and interests; for instance, we noted that many participants had infrequent or no drug-related police interactions, so we included questions to probe about why they thought this was the case.

The interviews were conducted over the phone or Zoom, depending on participants’ preferences, and lasted between 40 and 80 min. We encouraged participants to expand on responses, which guided the conversation. Interview audio recordings were transcribed verbatim by a professional transcriptionist, deidentified, and reviewed by the first author for accuracy. Memos were written by the first author throughout the research process as a reflective tool, beginning at the research design stage and continuing into the writing stage to aid in theme development and manuscript writing. This research received ethics approval from Simon Fraser University's Research Ethics Board (30001251).

### Data Analysis

We imported interview transcripts and memos into NVivo 14 (Lumivero, 2023) to help us organize the data and facilitate analysis. Grounded in an interpretive and social constructionist worldview, which aligns with our theoretical orientation, data were analyzed collaboratively by all authors using Clarke and Braun's (2022) reflexive thematic analysis. The first author read all the interview transcripts to familiarize herself with the data and then developed a list of codes that she discussed with the second author before conducting the first round of coding. First, we developed codes inductively based on overarching ideas, and then we developed codes deductively as we read through the data. We paid close attention to interview data that referred to police opinions or interactions, questioning how participants talked about themselves, others, and police in such conversations. We conducted two rounds of coding, followed by theme development. The analysis and development of themes continued into the writing process, reassessing, deleting, and creating themes or codes as necessary, in collaboration with all authors. In conducting the analysis, we found that our themes resonated with ideas from curriculum theory, and we used this lens to help understand and make sense of and frame our findings. The chosen qualitative approach helped develop implicit patterns or themes from the interviews, in line with the concepts borrowed from curriculum literature.

Because reflexivity was central to our analytic method, the first author maintained a personal journal to note questions, analytic ideas, and reflect on her positionality and how this shaped the research design and interpretation of the findings. The research team also met frequently to discuss the study, from design to analysis. Our research team was composed of the principal investigator (Alissa Greer), two doctoral students (Becca Wood and Naomi Zakimi) who worked as research coordinators, and a research collaborator with expertise in curriculum studies (Rodrigo Santos). We all currently work in academic settings and come from different academic, cultural, and socioeconomic backgrounds. We are all employed and stably housed; as such, we continually reflected and discussed how our own socioeconomic position shaped our analysis. We remained attentive to how our positions and experiences, similar to those of our participants, protected us from the impacts of criminalization. As such, each team member reflected on their own experiences with drug use, either privately through memoing or as a group when comfortable, to enrich our interpretation of the data.

### Demographic Characteristics

Table 1 shows participants’ demographic characteristics. Most participants identified as White (70%), and half identified as women. They primarily used cocaine (60%) and MDMA (ecstasy) (58%), followed by crack cocaine (20%) and heroin or fentanyl (20%); further, 85% of participants also used non-decriminalized drugs like hallucinogens and dissociatives. During the qualitative interviews, participants described their drug use in multiple ways, ranging from “recreational” or “therapeutic” to “problematic” or as an “addiction”; quantitatively, most of them used drugs between 1 and 10 days per month on average (72.5%), and 20% of them used drugs 21 days or more per month.

**Table 1.** Demographic Characteristics (N = 40).

Characteristic	*N* (%)
Gender	
Women	20 (50)
Men	17 (42.5)
Gender expansive	2 (5)
Prefer not to answer	1 (2.5)
Age	
18–30	13 (32.5)
31–40	15 (37.5)
41–50	11 (27.5)
51–60	1 (2.5)
Race/ethnicity	
White European	28 (70)
East Asian	5 (12.5)
Indigenous	1 (2.5)
South Asian	1 (2.5)
Southeast Asian	1 (2.5)
African	1 (2.5)
Latin American	1 (2.5)
Prefer not to answer	2 (5)
Health authority	
Vancouver	13 (32.5)
Interior British Columbia	10 (25)
Fraser	9 (22.5)
Vancouver Island	7 (17.5)
Northern British Columbia	1 (2.5)
Drugs used in the last month^a^	
Cocaine	24 (60)
MDMA/ecstasy	23 (57.5)
Crack cocaine	8 (20)
Heroin or fentanyl	8 (20)
Crystal meth/methamphetamine	6 (15)
Other (psychedelics, hallucinogenics, dissociatives, benzodiazepines, etc.)	34 (85)
Frequency of drug use	
1–10 days/month	29 (72.5)
11–20 days/month	2 (5)
21+ days/month	7 (17.5)
Prefer not to answer	2 (2.5)

^a^ Participants could report more than one substance.

## Findings

We developed three main themes (and related subthemes) related to what people who use drugs learn from the formal and hidden curriculum of decriminalization based on their discussions of police officers and policing (Table 2): (1) People like me do not get stopped by police: what the hidden curriculum teaches us about people who use drugs, (2) Decriminalization gives me peace of mind: what the formal curriculum of drug policy teaches us, and (3) Looking over the fence: the gap between the formal and hidden curriculum of drug policy. Taken together, these themes shed light on the way participants see themselves as different from “other” people who use drugs, underscoring the importance of social positioning and the privileges that come with it. The first theme explores the hidden curriculum through participants’ perceptions of and experiences with police officers, highlighting the messages that these participants receive and interpret about socially acceptable appearance and behavior. The second theme captures participants’ interpretations of the formal curriculum of decriminalization, which conveyed a sense of ease around police and greater acceptance of drug use. Finally, the third theme examines participants’ reflections on the policing of “other” people who use drugs; here, the disconnect between the formal curriculum of decriminalization and the hidden curriculum seen through policing practices reinforced the difference between themselves and “others” who were more likely to be policed. Importantly, we draw attention both to what is said and what is left unsaid and taken for granted, which also communicates ideas about participants and how they see themselves and other people who use drugs.

**Table 2.** Themes and Subthemes Developed from Interviews.

1. People like me do not get stopped by police: lessons from the hidden curriculum
1.1. Looking “normal:” what policing says about appearance
1.2. Fitting in: what policing says about appropriate or acceptable behavior
2*.* Decriminalization gives me peace of mind: what the formal curriculum of drug policy teaches us
3. Looking over the fence: the gap between the formal and hidden curriculum of drug policy

### People Like Me Do Not Get Stopped by Police: Lessons from the Hidden Curriculum

Participants had lived most of their lives and used drugs under a criminalization regime. However, less than half of the sample had any past drug-related contact with police, and they rarely recounted negative experiences or consequences from such encounters. In our conversations about the topics of decriminalization and policing drugs, all participants were asked about drug-related police contact since decriminalization; no participants reported drug-related police interactions within that time frame (one of 40 participants reported an unrelated police interaction since decriminalization due to a domestic dispute). Given the nature of our semi-structured question guide, not all participants were asked the same questions or had the opportunity to disclose past police interactions prior to decriminalization. However, 25 participants reported any sort of police contact (drug- or non-drug-related) before decriminalization; of those, 18 said that at least one of these interactions was drug-related, while 17 explicitly mentioned never having drug-related police contact. Participants reflected on their infrequent or lack of police contact, often attributing it to their “normal” physical appearance and “acceptable” behavior. Such reflections articulated a hidden curriculum of drug policy that implicitly positioned participants as specific kinds of subjects or people—specifically, how they were different from other people who use drugs who are relatively more marginalized and criminalized. In the following subthemes, we examine this hidden curriculum and its messages in greater depth, including how this lack of contact produced people who use drugs differently based on appearance and behavior.

#### Looking “Normal”: What Policing Says About Appearance

Across the sample, participants repeatedly attributed a lack of police contact to their physical appearance (e.g., looking “clean” and “well-dressed,” women looking more “feminine”), revealing how the hidden curriculum of drug policy reinforced stigmatizing distinctions between people who use drugs based on social class and ethnicity. The ability to look or present a certain way, such as dressing “well” or being “clean,” had to do with participants’ access to material and symbolic resources; limited opportunities, income, time, and space mean that presenting in the ways described by participants here is not a matter of choice, and, even when resources are available, people who are racialized simply do not have the option of being “invisible” to police.

For instance, Participant 1 conveyed a sense of invisibility to police attention attributed to appearing “normal”: “They wouldn’t stop someone like me randomly […] ‘cause I look very normal.” (Participant 1). In this quote, Participant 1 understood this lack of contact as emerging from a difference in how they looked and presented compared to people who come into contact with police; the latter were consequently constructed as *ab*normal. Such narratives illuminated that police action and inaction sent a message about social positioning and acceptance implicit in the hidden curriculum.

Among the few participants who had contact with police, the outcomes of police encounters were interpreted based on the social positioning conveyed through their physical appearance, as implied in the following quotes:

I’ve never had a negative police interaction for carrying drugs or buying drugs […] I look clean enough, I guess. I don’t look like I’m unhoused. (Participant 31)

I don’t think the cop is going to pick on me. If I go to a road stop, I’ve been to so many road stops that they just smell your breath and let you go, and I’m a pretty girl that looks well kept. (Participant 7)

These participants’ interpretations of police encounters were shaped less by their actual drug use and more by the social signals conveyed through their physical appearance, as pointed out by Participant 31. Specifically, they believed their appearance aligned with normative expectations of orderliness, cleanliness, and—for women—femininity, qualities implicitly associated with social belonging. Further, in naming what they were (“clean”) and what they were not (not “unhoused”), participants also took for granted and constructed other people who use drugs who came into contact with police as *not* clean, *not* well kept, and unhoused. In doing so, they differentiated themselves from “other” more marginalized individuals, distancing themselves from stigmatized notions of drug use, and, in turn, reproducing such distinctions, both through what they said and what they left unsaid.

Related to physical appearance, some participants also perceived race and ethnicity as reasons for police attention, again highlighting a hidden curriculum of drug policy that created different policing experiences among people who use drugs. Specifically, being White was described as a privilege that allowed people to either avoid police altogether or receive better treatment from police:

I would say I’m lucky because I come from a privileged demographic. And so I think the consequences of my drug use behavior have been less on me because of that. So I’m blonde, I’m white. And it puts me in kind of a privileged demographic. When I have, say, come in contact with the police because of my behaviour I feel like I’ve been much more likely to be let go or just let off. (Participant 5)

The message communicated by policing practices was clear for this participant: police attention and negative outcomes were reserved for people who use drugs who were, in this case, racialized. Being White offered some participants a privilege over racialized people who use drugs. Those who identified as White and privileged were easily able to occupy a normative non-drug user position as infrequent police contact enabled such subjectification; these participants were rarely confronted with the stigma and legal and social consequences of their drug use.

Therefore, even when participants reflected on and acknowledged their privilege, policing practices continued to send a message that defined “problematic” people who use drugs as deserving of continued criminalization while positioning others as everyday “mainstream” citizens. Ultimately, this perceived focus of police on marginalized populations served to reinforce participants’ perception of themselves as different from other people who use drugs, both through what they explicitly said about themselves and others and what they left unsaid.

#### Fitting in: What Policing Says About Appropriate or Acceptable Behavior

Closely tied to appearance, some participants also linked their invisibility to police attention to their behavior, suggesting that policing practices conveyed implicit lessons about what constitutes “appropriate” and “inappropriate” behavior or conduct, separating people who use drugs who are stopped by police from those who are not. Participants described how certain behaviors, particularly those perceived as visibilizing drug use, chaotic, or disruptive in public spaces, were more likely to elicit public complaints and police intervention. As one participant explained, “I’m not often doing chaotic things in the world that are, like, visible to people to be, like, calling the cops about” (Participant 27). Here, visibility is not solely related to drug consumption but also to behaviors framed as social disorder that were associated with drug use and seen as deserving of police attention, such as “blocking” the entrance to private businesses (Participant 4) or “collaps[ing] on the side of the street” (Participant 36). Although, once again, participants’ ability to not use drugs in view of the public and to choose private (e.g., own house) or semi-private spaces (e.g., festival and house party) had to do with their socioeconomic position. There was an implicit lesson about the type of behavior that was criminalized for people who use drugs, shaping more privileged people who use drugs as different.

Some participants explicitly conveyed their own abilities to maintain personal and professional responsibilities alongside ongoing substance use and contrasted this with others who were seen as “non-functioning,” again reinforcing the idea that people who use drugs have the ability to attract or avoid police action by controlling their behavior and abiding by neoliberal expectations of what a “functional” citizen ought to do. Some participants rationalized their lack of police contact by referencing their productivity and responsibility (e.g., having a stable job and paying taxes). For instance, Participant 28 explained that there are differences between people who “get caught with drugs”:

I don’t think I would [get caught with drugs] because you would never think by meeting me or seeing me, “oh, wow, she does drugs.” Or “she does that.” Or “she does—,” you know what I mean? You can tell the difference between someone who doesn’t do it recreationally, someone who is a functional addict, and then someone who is a homeless addict in a bad, bad, bad state. (Participant 28)

Failing to “function” and being “a homeless addict” are, for this participant, reasons for “getting caught with drugs.” These ideas question the citizenship of people who use drugs who cannot “function”; it teaches participants that people are citizens when and if they can fully participate in society as expected (hiding drug use, using in specific contexts, looking a certain way, etc.). What police officers attend to and do *not* attend to, therefore, shapes our worldview, makes available specific subject positions, and reinforces the existing social order.

Ultimately, the ideas presented in this theme are closely connected to neoliberal notions of functionality, productivity, responsibility, and order (Chatwin & Alexander, 2025; Fraser, 2004; Pereira & Carrington, 2015). Participants’ association of “chaos” with drug use further stigmatizes the position of people who use drugs and constructs order in narrow ways, obscuring the possibility that “order” can take on many forms (Fraser & Moore, 2008). Further, ideas of “chaotic behavior” echo ableist stereotypes about mental illness, which is often associated with violence and unpredictability among the public and in media discourse (Corrigan et al., 2009; Klin & Lemish, 2008). The emphasis on being a “functioning” member of society centres individual responsibility and choice as a main reason for limited police contact shows how, even under decriminalization, policing practices and the drug policies that are partly enacted through them cannot be decoupled from broader social and economic logics.

### Decriminalization Gives Me Peace of Mind: What the Formal Curriculum of Drug Policy Teaches Us

The formal “curriculum” of decriminalization dictated that police officers could no longer arrest or charge people for simple possession of specific drugs weighing under 2.5 g. Some participants directly spoke about the impact of this policy on them personally, mainly speaking about a “sense of relief” or “peace of mind” to possess illegal drugs. The policy reassured participants that they would not be penalized if they were in contact with police, demonstrating a sense of trust in the formal policy curriculum. Thus, despite most participants’ infrequent contact with police, being aware of the decriminalization policy or “curriculum” relieved anxiety and fear of police attention as well as the negative outcomes associated with it. Participant 1 described the feeling that decriminalization provided:

[Decriminalization] makes [potential police interactions] less nerve-wracking, that's for sure. It is nerve-wracking getting caught by the cops and then you have to deal with them, especially as an adult now. It’d feel a little—I wouldn’t really enjoy it. (Participant 1)

The decriminalization curriculum gave some people who use drugs a renewed sense of security in knowing the formal policy exists and believing that police officers would know about and practice the new policy. In such narratives, the formal “curriculum” of drug policy matters: whereas criminalization suggested that they were doing something wrong, *de*criminalization reinforced and legitimized the non-criminalized subject position for people who use drugs who were already able to inhabit such position due to their social privilege relative to marginalized, unhoused, poor, and racialized people who use drugs.

The formal curriculum of drug policy also sent a message about how participants would be policed; that they were no longer “breaking the law” provided relief for some participants who rarely encountered police:

[Interacting with police officers] would be even easier now because now—if I would be carrying drugs with me I wouldn’t even break the law. And then that makes it even relaxed—to interact with them, if I would ever be in a situation where a police officer approaches me in that way. And then I have drugs with me then that's—to be honest with you what changed for me since this law came out is, like, now that I don’t break the law anymore, it is relieving. […] Even though I’m already in a very privileged situation towards taking drugs and all of that and also towards police, but I think it's—even for me I can feel a little [exhales] relief. (Participant 3)

The idea that police officers could not charge participants with a crime anymore was also significant for participants because many of them actively hid that they used drugs from their social circle. Under criminalization and prohibitionist policies, which had dominated most of their lives, they had constituted themselves as “potentially” criminal. Therefore, the mere awareness of decriminalization reduced their fears and offered some protection against being marked by a criminal record, which they understood as a powerful mechanism of social exclusion, particularly in relation to employers and some friends or family members:

If that policy [decriminalization] didn’t exist it's also, like, what is the state going to gain by criminalizing me, right? If I get—if I have a bag in my pocket and I get charged, then I couldn't [inaudible], I’d lose my job, lose my—I’d lose my livelihood, I’d lose my house. I lose social connections so then, like, it's just a very selfish state view. Why would—what would the state gain, you know what I mean? (Participant 4)

And that's where it's [decriminalization is] helpful because otherwise you have to tell them [potential employers]: “I was arrested.” It's on your record. If you go for your job and people look into it, it's there. So it's, like, you know what I mean? It comes out. Something like that can come out and it's fucked. That's 10 or 11 people's lives that have all been black marked because of what? A gram of weed or a gram of coke? That's ridiculous. And it's, like, I don’t know. It's so nuts to think that a friend of mine couldn’t go see his family in the States ‘cause he's not allowed back in the States any more. […] That's because he had a gram and a half of cocaine 12 years ago. (Participant 6)

As shown in these quotes, decriminalization allowed participants to keep their drug use hidden by not being “outed” by a criminal record—a “mark” or label that may jeopardize their social position. The formal message sent by decriminalization carried symbolic weight and was important to how they constituted themselves as drug users, serving not only as legal protection but as a form of legitimacy, as it provided a sense of security and reduced the risk of being socially or professionally discredited because of their drug use. Thinking about hypothetical interactions with police was less concerning after decriminalization. However, even though this was seen as a benefit of decriminalization for these participants, it speaks to the deeply ingrained fear of being “outed” that continues to force people to hide their drug use, as well as to the continued marginalization of people who use drugs who suffer the brunt of criminalization.

### Looking Over the Fence: The Gap Between the Formal and Hidden Curriculum of Drug Policy

As shown in the previous themes, participants were aware of negative police interactions among “other” people who use drugs, even in the context of the “formal” curriculum of decriminalization, which, as written, meant that police officers should refrain from criminalizing drug use. However, the contrast between Themes 1 and 2 shows a clear division between the formal curriculum of decriminalization (and drug policy in general) and the hidden curriculum enacted through police action *for certain groups* of people who use drugs; only some people who use drugs benefited or experienced meaningful change from drug policy. Awareness of this difference in police practices, in and of itself, implicitly legitimized specific kinds of drug user subjects, thereby dividing participants from more criminalized (and often marginalized) people who use drugs. This gap was illustrated through participants’ repeated narratives about the experiences of people who use drugs who did not look or act like them, creating a sense of “looking over the fence” at (and producing) the criminalized “other.”

Awareness of how others were treated by police shaped participants’ understanding of their own social position and its protective effects. This awareness often produced a sense of insecurity and, at times, shame that they could personally be policed and criminalized if it were not for their current social position. The importance of this social position was articulated or “taught” to participants through the “hidden” curriculum of drug policy, not only based on their own experiences with police officers (or lack thereof), but based on how other people who use drugs were treated by police too. Awareness of this gap created a sense of insecurity and hesitancy about police officers’ intentions:

When I was young, I used to look up to them [police officers] and think oh, these are real paragons. But instead I just see a lot of just high-school bullies almost in terms of their attitudes and how they act. And again, have I had positive and supportive interactions with police? Yes, but few. Have I experienced or heard of negative ones? Far more. (Participant 13)

This participant exemplifies how they have experienced or “heard of” unjust police behavior over the years, showing growing skepticism shaped by both direct and indirect experiences. There is concern with how police behave: they are described as “high-school bullies,” suggesting that power abuses generate fear and serve as a warning to avoid being labelled and constituted as the “other” category of people who use drugs. The negative anecdotal and personal experiences with police contrast with their original belief of police as “real paragons” whom they admired and point to the perceived gap between what they expect of police officers and officers’ actual practice. The contrast between early admiration and later disillusionment illustrated the gap between the expected role of police officers and lived or observed practice. Participant 39 specifically shows the dissonance created from the incongruency between personal experiences and other people's stories about policing:

I haven’t had any bad—too bad, like, negative attention that's been focused towards me personally, thank God, knock on wood. But I’ve definitely heard stories where it's, like, the police did not behave in a way that was, like, whoa, like, do they not know their own laws? Or do they not know about resources or haven’t they had any non-violent crisis intervention training? (Participant 39)

By questioning police officers’ understanding and application of the law (“do they not know their own laws?”), Participant 39 suggests that *some* police officers do not follow the formal curriculum based on their knowledge of other people's experiences. This perceived unpredictability in how laws are applied produced a sense of insecurity, even for those who had limited personal contact with police and under drug decriminalization, because it reinforced the idea that policing practices could be arbitrary and based on social markers like class and ethnicity rather than drug use or possession. Many participants were concerned particularly with stories and experiences they had seen or heard about structurally vulnerable people who use drugs: “I think often when police encounter people who use drugs I think it's often encounters with marginalized people” (Participant 4); “I think they will continue to criminalize poverty” (Participant 25). Many still held this belief despite decriminalization:

I can hide my supply in my house. But technically, what I’m doing is illegal. But yeah, suffice to say is, like, I’ve seen it [decriminalization] impact people that I work with quite closely in a negative way 'cause I’ve seen an increase of police. (Participant 20)

This participant worked with other people who use drugs and had experienced firsthand the differences in police treatment for people in positions vulnerable to police contact before and after decriminalization. These insights reflect a broader understanding of the hidden curriculum of drug policy, which communicates that drug policies can be applied selectively, continuing to surveil and punish people who are already marginalized despite the formal protections of decriminalization.

As this theme shows, some participants struggled to reconcile their expectations of police officers under decriminalization (the formal curriculum) with the divergent experiences (particularly those of others), resulting in conflicting feelings about police officers: “I have a love and hate… I shouldn’t say hate, hate is a strong word— irritation with the police” (Participant 33). The influence of stories about police interactions with people who use drugs was powerful, showing that we can learn about ourselves, others, policing, and drug policy through indirect experiences. Ultimately, knowledge of others’ experiences with police produced a fear of being discovered and constituted as a specific kind of drug user (irresponsible, “chaotic,” non-functioning, or otherwise stigmatized); awareness of the gap between the hidden and formal curriculum of drug policy, and how this gap could potentially affect them, created conflicting perceptions of police. Participants’ limited personal encounters with police did not erase their awareness of broader systemic issues. Rather, they learned through observation and anecdotal accounts that privilege mediates how the curriculum of drug policy is enacted and experienced. The hidden curriculum thus continues to constrain how people who use drugs see and position themselves, instill fear, and reinforce structural inequities, even under a legal framework intended to reduce harm.

## Discussion

This study examined how people who use drugs make sense of drug decriminalization and policing in B.C., with a focus on their social position. Participants’ narratives illuminated a formal curriculum, reflected in the written policy, and a “hidden” curriculum shaped by how police acted (or failed to act). The formal curriculum provided protection from criminalization and a sense of relief, as it allowed participants to position themselves as members of the majority social group by alluding to notions of responsibility and control. However, participants also perceived a disconnect between policy and police practices. Police officer behavior and decision-making about who to stop or target sent implicit messages about whose drug use was acceptable and whose was not. These distinctions shaped how participants saw themselves in relation to more visibly marginalized people who use drugs, reinforcing boundaries between them. Our findings also highlight the fragility and instability of participants’ identities when policies are applied unevenly. Overall, these findings suggest that the formal policy and its enforcement through policing matter for how people understand and position themselves and others.

In our study, the formal curriculum of decriminalization as enacted by police officers conveyed lessons that shaped and constrained how people who use drugs saw and positioned themselves; specifically, people who use drugs in our sample experienced a sense of relief or eased anxiety based on their awareness of decriminalization. This formal curriculum provided reassurance around police as well as less fear of being “outed” and the social ramifications of being publicly identified as a drug user. This relief came from knowledge that police officers could not arrest or charge them; even if such an encounter was rare amongst our sample, the law “on the books” or the formal curriculum mattered for them. Decriminalization changed the role of police “on paper,” and that change had meaning beyond its application in practice by providing a sense of relief and security.

In contrast to the formal curriculum, the hidden curriculum of drug policy revealed a precarity to decriminalization. The hidden curriculum—or what is not explicitly outlined in the formal drug policy curriculum (Meares & Prowse, 2021)—was conveyed through beliefs and experiences of “who” benefits from decriminalization itself. Observations of policing provided lessons about social positioning and social order based on who and what police attended to, producing *some* people who use drugs as “functioning” and law-abiding in contrast with people who use drugs who were perceived as uncontrolled, irresponsible, and marginalized due to their social position. Participants’ own experiences, coupled with second-hand knowledge of police discrimination and power abuses, communicated how social position matters; decriminalization could not be trusted by *all* people who use drugs. Previous studies with recreational drug users echo how this social group can perceive themselves as different from other people who use drugs, often evoking notions of being more “in control” and “functioning” in line with a neoliberal rationale (Askew & Salinas, 2019; Decorte, 2001).

Our findings also complement social identity literature, which suggests that people who use drugs seek belonging in an in-group in comparison with out-groups. For instance, a questionnaire study conducted with participants recruited from universities and party scene websites in Melbourne, Australia found that many identified with a “party drug user” social identity; stronger identification was associated with fewer drug-related “problems” (e.g., anxiety, memory loss, psychosis, and physical harm), suggesting that social identity could be a protective factor by making people seek and share information about harm minimization group norms (Hynes & Zinkiewicz, 2007). In our study, participants similarly took on the responsible and functioning social position that was produced through the uneven enactment of drug policy; the structural order that allowed them to take on such a position “protected” them from police contact and criminalization.

Our findings further suggest that the differential treatment of people who use drugs by police officers may only reinforce ideas around “who” is deserving of the protections afforded by decriminalization. Our findings show that infrequent or benign interactions with police officers (which are echoed in previous studies of “socially included” people who use drugs; Askew & Salinas, 2019; Cruz, 2015; Hausser et al., 1999; Rödner, 2006) reinforced and made available a subject position as *different* from other, more marginalized, people who use drugs. Our study, therefore, shows the complex way in which people who use drugs come to understand and position themselves in ways that are reinforced through their experiences with and knowledge of police. The way that police enforced drug policy (or did not enforce it with some segments of the population) contributed to how participants saw themselves as people who use drugs. Using drugs was criminalized only for some people, and because of aspects that went beyond drug use or possession, regardless of the formal curriculum of drug policy.

Finally, our study provides a unique contribution to the literature by not only identifying a “gap” between what is written in policy and what is practiced through enforcement practices, but by unpacking how this gap can be a site of meaning-making and subject formation. Prior research has evidenced the disconnect between policy and practice, demonstrating that it can undermine public health objectives, increase health risks, and reproduce inequality (Beletsky et al., 2005; Burris et al., 2004; Burris et al., 2010; Swanson et al., 2006). Our study extends this work by using a curriculum lens to examine drug policy and policing to interrogate what it does socially. This lens allowed us to view policing practices as a type of informal education that “teaches” people who use drugs about themselves, others, and the world. The enforcement (or selective non-enforcement) of drug policy operationalizes a hidden curriculum of drug policy. Participants learned, through both personal experience and observation, that drug use is not criminalized uniformly; rather, its criminalization is contingent on social markers such as housing status, race, and perceived respectability. This disparity in enforcement communicated powerful, if implicit, lessons about whose behavior is tolerated and whose is punished, which ultimately shaped how participants understood their own position within the social and legal order. In short, our findings suggest that the gap between policy and practice, as enacted by police, has important consequences not only for public health and people who use drugs’ risk environments, but it can also shape discourse and subjects.

These findings have implications for both policy and research. The formal curriculum of decriminalization improved how people who use drugs in our sample felt in their everyday lives. They felt a sense of relief from the fear of being “marked” by a criminal record (Pager, 2003). For this demographic of people who are protected from policing and criminal justice system interactions, decriminalization may provide security and stability through the anonymity of drug use that is key to keeping their jobs and social lives intact, particularly where drug use stigma still exists. The message sent by the formal curriculum of drug policy matters to people who use drugs and should be carefully considered. Drug policies, and what they state in theory, can therefore be important even for those who are not in frequent contact with the criminal justice system.

However, we must be careful not to overestimate the importance of the formal curriculum of drug policy in the face of what actually happens “on the streets” and how police enact drug policy in practice, paying closer attention to the “hidden” curriculum. The latter, disclosed through police (in)action, has the power to shape and legitimate some people who use drugs over others based, ultimately, on notions that have to do with people's social position. Given that one of the goals of decriminalization was to reduce stigma (Government of British Columbia, 2022), officers’ application of the law and discretionary practices should be closely evaluated in times of policy change. In this case, the continued reliance on police to practice a policy, decriminalization, which is intended to move drug use out of the criminal realm, calls for critical reconsideration. This is particularly important given that the disconnect between policies and how they are enacted in practice can have effects beyond this specific moment in time; this gap can cause distrust in legal institutions and discourage engagement in formal democratic processes (Justice & Meares, 2014; Meares, 2016), as well as negatively impact people who use drugs’ health risk environment (Burris et al., 2004; Burris et al., 2010).

Yet, when the curricular gap is uncovered and reflected upon, it can also create opportunities for re-imagining drug policy and the role of the criminal justice system and people who use drugs. Uncovering and naming the hidden curriculum is valuable in and of itself (Apple, 1971), and we agree with Apple (2019) that “we have an ethical obligation to make public the effects of these policies” (p. 2). Examining what the hidden curriculum of drug policy teaches the public, and specifically people who use drugs, is important if we are to address inequity in policing practices. Reflecting on these issues, like many participants did, sheds light on policing differences and how subjects and identities are constructed in interactions with the criminal justice system, which can be an empowering exercise as it can foster a critical awareness of power and people's own social position (Giroux, 2001). Developing such critical awareness allows people to make choices about which ideas and messages they align with, how to participate in a democracy, and re-imagine the way they think about drugs, people who use drugs, and themselves.

This study is not without limitations. First, while we aimed to interview people from across different regions of British Columbia, we only interviewed one person from Northern British Columbia. Ensuring this population's perspective is included is important given that drug use stigma is generally higher in these regions, which could partly explain the difficulty in recruiting people who may fear being “outed” in their communities (Greer et al., 2019). Second, because of the importance of “hiding” that they use drugs, it is possible that participants did not feel comfortable expressing their full range of opinions about and experiences of police and drug use. Further, people who have had more negative experiences with police and involvement in the criminal justice system may have been less likely to participate in this study due to fear of being identified. Third, the focus on people who are housed and employed could raise concerns about reinforcing the development of inequitable policies that favour those who are already socially privileged. We acknowledge this focus as a limitation, and, as such, emphasize that our findings should hopefully draw attention to the differences in policing practices and to the power and need of “mak[ing] public the effects of […] policies” (Apple, 2019, p. 2) in line with our theoretical stance.

## Conclusion

The current study highlights how people who use drugs interpret and internalize messages that shape how they see and position themselves as such through their interactions with and views of police officers, who are often responsible for enacting drug policy curriculum. Overall, our findings deepen our understanding of decriminalization by providing nuance and context to how it was experienced in practice by a specific segment of the population. Importantly, we document how policing enactment of drug policy communicates ideas to and influences people who use drugs, even when they are not in frequent contact with the criminal justice system. Ultimately, reflecting on the formal and hidden curricula of drug policy can lead to a critical understanding of how such policy, and in turn policing, shapes our understandings and constructions of society and ourselves, sowing the seeds for action towards a more equity-oriented understanding and treatment of drugs and people who use drugs.

## References

[bibr1-00914509251396996] Aitken C. Moore D. Higgs P. Kelsall J. Kerger M. (2002). The impact of a police crackdown on a street drug scene: Evidence from the street. International Journal of Drug Policy, 13(3), 193–202. 10.1016/S0955-3959(02)00075-0

[bibr2-00914509251396996] Apple M. W. (1971). The hidden curriculum and the nature of conflict. Interchange, 2(4), 27–40. 10.1007/BF02287080

[bibr3-00914509251396996] Apple M. W. (2004). Ideology and curriculum (3rd ed.). Routledge Falmer.

[bibr4-00914509251396996] Apple M. W. (2019). On doing critical policy analysis. Educational Policy, 33(1), 276–287. 10.1177/0895904818807307

[bibr5-00914509251396996] Askew R. Salinas M. (2019). Status, stigma and stereotype: How drug takers and drug suppliers avoid negative labelling by virtue of their ‘conventional’ and ‘law-abiding’ lives. Criminology & Criminal Justice, 19(3), 311–327. 10.1177/1748895818762558

[bibr6-00914509251396996] Bacchi C. L. (2009). Analysing policy: what's the problem represented to be? Pearson.

[bibr7-00914509251396996] Ball S. J. (1994). Education reform: a critical and post structural approach. Open University Press.

[bibr8-00914509251396996] Ball S. J. (2003). The teacher's soul and the terrors of performativity. Journal of Education Policy, 18(2), 215–228. 10.1080/0268093022000043065

[bibr9-00914509251396996] Ball S. J. Maguire M. Braun A. (2012). How schools do policy: Policy enactments in secondary schools. Routledge.

[bibr10-00914509251396996] Beletsky L. Cochrane J. Sawyer A. L. Serio-Chapman C. Smelyanskaya M. Han J. Robinowitz N. Sherman S. G. (2015). Police encounters among needle exchange clients in Baltimore: Drug law enforcement as a structural determinant of health. American Journal of Public Health, 105(9), 1872–1879. 10.2105/ajph.2015.302681 26180948 PMC4539845

[bibr11-00914509251396996] Beletsky L. Macalino G. E. Burris S. (2005). Attitudes of police officers towards syringe access, occupational needle-sticks, and drug use: A qualitative study of one city police department in the United States. International Journal of Drug Policy, 16(4), 267–274. 10.1016/j.drugpo.2005.01.009

[bibr12-00914509251396996] Berger E. J. (2025). Dealing with privilege in a Nordic welfare state? How experiences with, and perceptions of, police, markets, and violence shape decision-making among affluent drug dealers. European Journal of Criminology, 22(1), 127–146. 10.1177/14773708241272737

[bibr13-00914509251396996] Bernstein B. (1996). Pedagogy, symbolic control, and identity: theory, research, critique. Taylor & Francis.

[bibr14-00914509251396996] Bowles J. M. Smith L. R. Mittal M. L. Harding R. W. Copulsky E. Hennessy G. Dunkle A. Davidson P. J. Wagner K. D. (2021). “I wanted to close the chapter completely… and I feel like that [carrying naloxone] would keep it open a little bit”: Refusal to carry naloxone among newly-abstinent opioid users and 12-step identity. International Journal of Drug Policy, 94, 103200. 10.1016/j.drugpo.2021.103200 33765517 PMC10155624

[bibr15-00914509251396996] Buckingham S. A. Frings D. Albery I. P. (2013). Group membership and social identity in addiction recovery. Psychology of Addictive Behaviors, 27(4), 1132–1140. 10.1037/a0032480 23586453

[bibr16-00914509251396996] Bungay V. Johnson J. L. Varcoe C. Boyd S. (2010). Women's health and use of crack cocaine in context: Structural and ‘everyday’ violence. International Journal of Drug Policy, 21(4), 321–329. 10.1016/j.drugpo.2009.12.008 20116989

[bibr17-00914509251396996] Burris S. Blankenship K. M. Donoghoe M. Sherman S. Vernick J. S. Case P. Lazzarini Z. Koester S. (2004). Addressing the “risk environment” for injection drug users: The mysterious case of the missing cop. The Milbank Quarterly, 82(1), 125–156. 10.1111/j.0887-378X.2004.00304.x 15016246 PMC2690204

[bibr18-00914509251396996] Burris S. Wagenaar A. C. Swanson J. Ibrahim J. K. Wood J. Mello M. M. (2010). Making the case for laws that improve health: A framework for public health law research. The Milbank Quarterly, 88(2), 169–210. 10.1111/j.1468-0009.2010.00595.x 20579282 PMC2980343

[bibr19-00914509251396996] Chatwin C. Alexander R. G. (2025). Virtuous drug use in the neoliberal age. Drugs: Education, Prevention and Policy, 32(4), 1–9. 10.1080/09687637.2024.2443659 40206199

[bibr20-00914509251396996] Clarke V. Braun V. (2022). Thematic analysis: A practical guide. Sage Publications.

[bibr21-00914509251396996] Corrigan P. W. Kuwabara S. A. O'shaughnessy J. (2009). The public stigma of mental illness and drug addiction: Findings from a stratified random sample. Journal of Social Work, 9(2), 139–147. 10.1177/1468017308101818

[bibr22-00914509251396996] Cruz O. S. (2015). Nonproblematic illegal drug use: Drug use management strategies in a Portuguese sample. Journal of Drug Issues, 45(2), 133–150. 10.1177/0022042614559842

[bibr23-00914509251396996] David J.-D. Mitchell M. (2021). Contacts with the police and the over-representation of indigenous peoples in the Canadian criminal justice system. Canadian Journal of Criminology and Criminal Justice, 63(2), 23–45. 10.3138/cjccj.2020-0004

[bibr24-00914509251396996] DeBeck K. Cheng T. Montaner J. S. Beyrer C. Elliott R. Sherman S. Wood E. Baral S. (2017). HIV and the criminalisation of drug use among people who inject drugs: A systematic review. The Lancet HIV, 4(8), e357–e374. 10.1016/S2352-3018(17)30073-5 PMC6005363 28515014

[bibr25-00914509251396996] Decorte T. (2001). Drug users’ perceptions of ‘controlled’ and ‘uncontrolled’ use. International Journal of Drug Policy, 12(4), 297–320. 10.1016/S0955-3959(01)00095-0

[bibr26-00914509251396996] Department of Justice. (2024). *Understanding the Overrepresentation of Indigenous people in the Criminal Justice System*. Government of Canada. Retrieved November 14 from https://www.justice.gc.ca/socjs-esjp/en/ind-aut/uo-cs

[bibr27-00914509251396996] Dertadian G. C. Askew R. (2024). Towards a social harm approach in drug policy. International Journal of Drug Policy, 127, 104425. 10.1016/j.drugpo.2024.104425 38615484

[bibr28-00914509251396996] Fraser S. (2004). ‘It's your life!': Injecting drug users, individual responsibility and hepatitis C prevention. Health (London), 8(2), 199–221. 10.1177/1363459304041070 15068637

[bibr29-00914509251396996] Fraser S. Moore D. (2008). Dazzled by unity? Order and chaos in public discourse on illicit drug use. Social Science & Medicine, 66(3), 740–752. 10.1016/j.socscimed.2007.10.012 18006200

[bibr30-00914509251396996] Fraser S. Pienaar K. Dilkes-Frayne E. Moore D. Kokanovic R. Treloar C. Dunlop A. (2017). Addiction stigma and the biopolitics of liberal modernity: A qualitative analysis. International Journal of Drug Policy, 44, 192–201. 10.1016/j.drugpo.2017.02.005 28366599

[bibr31-00914509251396996] Friedman J. Syvertsen J. L. Bourgois P. Bui A. Beletsky L. Pollini R. (2021). Intersectional structural vulnerability to abusive policing among people who inject drugs: A mixed methods assessment in California's central valley. International Journal of Drug Policy, 87, 102981. 10.1016/j.drugpo.2020.102981 33129133 PMC7940555

[bibr32-00914509251396996] Giroux H. (1981). Ideology, culture, and the process of schooling. Temple University Press.

[bibr33-00914509251396996] Giroux H. A. (2001). Theory and Resistance in Education: Towards a Pedagogy for the Opposition. ABC-CLIO, LLC. https://books.google.ca/books?id=g33rOgAACAAJ

[bibr34-00914509251396996] Giroux H. A. Penna A. N. (1979). Social education in the classroom: The dynamics of the hidden curriculum. Theory & Research in Social Education, 7(1), 21–42. 10.1080/00933104.1979.10506048

[bibr35-00914509251396996] Government of British Columbia. (2022). *Decriminalizing people who use drugs in B.C. - Province of British Columbia*. https://www2.gov.bc.ca/gov/content/overdose/decriminalization

[bibr36-00914509251396996] Government of Canada. (2021). About the Good Samaritan Drug Overdose Act. https://www.canada.ca/en/health-canada/services/opioids/about-good-samaritan-drug-overdose-act.html

[bibr37-00914509251396996] Gramsci A. (1971). Selections from the prison notebooks of Antonio Gramsci. International Publishers.

[bibr38-00914509251396996] Greer A. Selfridge M. Card K. Benoit C. Jansson M. Lee Z. Macdonald S. (2022a). Factors contributing to frequent police contact among young people: A multivariate analysis including homelessness, community visibility, and drug use in British Columbia, Canada. Drugs: Education, Prevention and Policy, 29(2), 168–174. 10.1080/09687637.2021.1872500

[bibr39-00914509251396996] Greer A. Selfridge M. Watson T. M. Macdonald S. Pauly B. (2021). Young people who use drugs views toward the power and authority of police officers. Contemporary Drug Problems, 49(2), 170–191. 10.1177/00914509211058989 35465248 PMC9021434

[bibr40-00914509251396996] Greer A. Zakimi N. Butler A. Ferencz S. (2022b). Simple possession as a ‘tool’: Drug law enforcement practices among police officers in the context of depenalization in British Columbia, Canada. The International Journal of Drug Policy, 99, 103471–103471. 10.1016/j.drugpo.2021.103471 34600414

[bibr41-00914509251396996] Greer A. M. Amlani A. Burmeister C. Scott A. Newman C. Lampkin H. Pauly B. Buxton J. A. (2019). Peer engagement barriers and enablers: Insights from people who use drugs in British Columbia, Canada. Canadian Journal of Public Health, 110(2), 227–235. 10.17269/s41997-018-0167-x 30610564 PMC6964581

[bibr42-00914509251396996] Hausser D. Kübler D. Dubois-Arber F. (1999). Characteristics of heroin and cocaine users unknown to treatment agencies. Results from the Swiss hidden population study. Sozial- Und Praventivmedizin, 44(5), 222–232. 10.1007/BF01341495 10588038

[bibr43-00914509251396996] Hynes A. Zinkiewicz L. (2007). A social identity approach to party drug use and associated harm minimisation. In *Psychology making an impact: Proceedings of the 42nd Australian Psychological Society Annual Conference*.

[bibr44-00914509251396996] Jackson P. W. (1968). Life in classrooms. Holt, Rinehart and Winston.

[bibr45-00914509251396996] Jackson P. W. (1992). Conceptions of curriculum and curriculum specialists. In Jackson P. W. (Ed.), Handbook of Research on Curriculum (pp. 3–40). Macmillan.

[bibr46-00914509251396996] Justice B. (2017). Curriculum theory and the welfare state. Espacio, Tiempo y Educación, 4(2), 19–41. 10.14516/ete.183

[bibr47-00914509251396996] Justice B. Meares T. L. (2014). How the criminal justice system educates citizens. The Annals of the American Academy of Political and Social Science, 651(1), 159–177. 10.1177/0002716213502929

[bibr48-00914509251396996] Kerr T. Small W. Wood E. (2005). The public health and social impacts of drug market enforcement: A review of the evidence. International Journal of Drug Policy, 16(4), 210–220. 10.1016/j.drugpo.2005.04.005

[bibr49-00914509251396996] Klin A. Lemish D. (2008). Mental disorders stigma in the media: Review of studies on production, content, and influences. Journal of Health Communication, 13(5), 434–449. 10.1080/10810730802198813 18661386

[bibr50-00914509251396996] Latimore A. D. Bergstein R. S. (2017). “Caught with a body” yet protected by law? Calling 911 for opioid overdose in the context of the good samaritan law. International Journal of Drug Policy, 50, 82–89. 10.1016/j.drugpo.2017.09.010 29040841

[bibr51-00914509251396996] Loader I. Mulcahy A. (2003). Policing and the condition of England: Memory, politics and culture. Oxford University Press.

[bibr52-00914509251396996] Lumivero. (2023). *NVivo (Version 14)*. www.lumivero.com

[bibr53-00914509251396996] Meares T. (2016). Policing and procedural justice: Shaping citizens’ identities to increase democratic participation. Northwestern University Law Review, 111(6), 1525–1535.

[bibr54-00914509251396996] Meares T. Prowse G. (2021). Policing as public good: Reflecting on the term “to protect and serve” as dialogues of abolition. Florida Law Review, 73(1), 1–30.

[bibr55-00914509251396996] Meng Y. (2017). Profiling minorities: Police stop and search practices in Toronto, Canada. Human Geographies: Journal of Studies & Research in Human Geography, 11(1), 5–23. 10.5719/hgeo.2017.111.1

[bibr56-00914509251396996] Moore D. Fraser S. (2006). Putting at risk what we know: Reflecting on the drug-using subject in harm reduction and its political implications. Social Science & Medicine, 62(12), 3035–3047. 10.1016/j.socscimed.2005.11.067 16413645

[bibr57-00914509251396996] Neve H. Collett T. (2018). Empowering students with the hidden curriculum. The Clinical Teacher, 15(6), 494–499. 10.1111/tct.12736 29178606

[bibr58-00914509251396996] Pager D. (2003). The mark of a criminal record. American Journal of Sociology, 108(5), 937–975. 10.1086/374403

[bibr59-00914509251396996] Pereira M. Carrington K. (2015). Irrational addicts and responsible pleasure seekers: Constructions of the drug user. Critical Criminology, 24(3), 379–389. 10.1007/s10612-015-9298-z

[bibr60-00914509251396996] Perrin S. Bertrand K. Langlois E. (2021). Avoiding the stigma. A qualitative study of socially included women's experiences of drug use and dealing, health services and the police in France. International Journal of Drug Policy, 87, 102850. 10.1016/j.drugpo.2020.102850 32665146

[bibr61-00914509251396996] Pickard H. (2021). Addiction and the self. Noûs, 55(4), 737–761. 10.1111/nous.12328

[bibr62-00914509251396996] Pinar W. F. Reynolds W. M. Slattery P. Taubman P. M. (1995). Understanding curriculum: An introduction to the study of historical and contemporary curriculum discourses. Peter Lang Publishing, Inc.

[bibr63-00914509251396996] Popkewitz T. S. (1998). Struggling for the Soul. The Politics of Schooling and the Construction of the Teacher. Teachers College Press.

[bibr64-00914509251396996] Ritter A. (2021). Analysing the discursive effects of drug policy. In *Drug Policy* (pp. 89–103). Routledge.

[bibr65-00914509251396996] Rodner S. (2005). “I am not a drug abuser, I am a drug user": A discourse analysis of 44 drug users’ construction of identity. Addiction Research & Theory, 13(4), 333–346. 10.1080/16066350500136276

[bibr66-00914509251396996] Rödner S. (2006). Practicing risk control in a socially disapproved area: Swedish socially integrated drug users and their perception of risks. Journal of Drug Issues, 36(4), 933–951. 10.1177/002204260603600408

[bibr67-00914509251396996] Rose N. S. (1999). Governing the soul: the shaping of the private self (2nd ed.). Routledge.

[bibr68-00914509251396996] Sarang A. Rhodes T. Sheon N. Page K. (2010). Policing drug users in Russia: Risk, fear, and structural violence. Substance Use & Misuse, 45(6), 813–864. 10.3109/10826081003590938 20397872 PMC2946846

[bibr69-00914509251396996] Selfridge M. Mitchell L. Greer A. Macdonald S. Pauly B. (2020). “Accidental intimacies”: Reconsidering bodily encounters between police and young people who use drugs. Contemporary Drug Problems, 47(3), 231–250. 10.1177/0091450920929101

[bibr70-00914509251396996] Sibley A. L. Baker R. Levander X. A. Rains A. Walters S. M. Nolte K. Colston D. C. Piscalko H. M. Schalkoff C. A. Bianchet E. (2023). “I am not a junkie”: Social categorization and differentiation among people who use drugs. International Journal of Drug Policy, 114, 103999. 10.1016/j.drugpo.2023.103999 36905779 PMC10066877

[bibr71-00914509251396996] Swanson J. Burris S. Moss K. Ullman M. (2006). Justice disparities: Does the ADA enforcement system treat people with psychiatric disabilities fairly. Maryland Law Review, 66(1), 94–139.

[bibr72-00914509251396996] Tajfel H. Turner J. C. (2004). The social identity theory of intergroup behavior. In *Political psychology* (pp. 276–293). Psychology Press.

[bibr73-00914509251396996] Tyler I. Slater T. (2018). Rethinking the sociology of stigma (Vol. 66, pp. 721–743). Sage Publications Sage UK.

[bibr74-00914509251396996] Tyler R. W. (1974). Basic principles of curriculum and instruction. University of Chicago Press.

[bibr75-00914509251396996] van der Meulen E. Chu S. K. H. Butler-McPhee J. (2021). “That's why people don't call 911”: Ending routine police attendance at drug overdoses. International Journal of Drug Policy, 88, 103039. 10.1016/j.drugpo.2020.103039 33227637

[bibr76-00914509251396996] Wacquant L. (2009). Punishing the poor. Duke University Press.

[bibr77-00914509251396996] Walker S. Seear K. Higgs P. Stoove M. Wilson M. (2020). “A spray bottle and a lollipop stick": An examination of policy prohibiting sterile injecting equipment in prison and effects on young men with injecting drug use histories. International Journal of Drug Policy, 80, 102532. 10.1016/j.drugpo.2019.07.027 31427211

[bibr78-00914509251396996] Werb D. Wood E. Small W. Strathdee S. Li K. Montaner J. Kerr T. (2008). Effects of police confiscation of illicit drugs and syringes among injection drug users in Vancouver. International Journal of Drug Policy, 19(4), 332–338. 10.1016/j.drugpo.2007.08.004 17900888 PMC2529170

[bibr79-00914509251396996] Wood E. F. Werb D. Beletsky L. Rangel G. Cuevas Mota J. Garfein R. S. Strathdee S. A. Wagner K. D. (2017). Differential experiences of Mexican policing by people who inject drugs residing in Tijuana and San Diego. International Journal of Drug Policy, 41, 132–139. 10.1016/j.drugpo.2016.12.010 28111221 PMC5342893

[bibr80-00914509251396996] Xavier J. Greer A. Crabtree A. Ferencz S. Buxton J. A. (2021). Police officers’ knowledge, understanding and implementation of the Good Samaritan Drug Overdose Act in BC, Canada. International Journal of Drug Policy, 97, 103410. 10.1016/j.drugpo.2021.103410 34438275

[bibr81-00914509251396996] Xavier J. Greer A. Pauly B. Loyal J. Mamdani Z. Ackermann E. Barbic S. Buxton J. A. (2022). “There are solutions and I think we're still working in the problem”: The limitations of decriminalization under the good Samaritan drug overdose act and lessons from an evaluation in British Columbia, Canada. International Journal of Drug Policy, 105, 103714. 10.1016/j.drugpo.2022.103714 35561485

[bibr82-00914509251396996] Zakimi N. Greer A. Butler A. (2022). Too many hats? The role of police officers in drug enforcement and the community. Policing: A Journal of Policy and Practice, 16(4), 615–629. 10.1093/police/paab082

[bibr83-00914509251396996] Zampini G. F. Buck-Matthews E. Killick A. Salter L. (2021). We, ourselves and us: Tensions of identity, intersubjectivity and positionality stemming from the people and dancefloors project. International Journal of Drug Policy, 98, 103096. 10.1016/j.drugpo.2020.103096 33446396

